# Cost-Effectiveness of One Year Dementia Follow-Up Care by Memory Clinics or General Practitioners: Economic Evaluation of a Randomised Controlled Trial

**DOI:** 10.1371/journal.pone.0079797

**Published:** 2013-11-25

**Authors:** Els Meeuwsen, René Melis, Geert van der Aa, Gertie Golüke-Willemse, Benoit de Leest, Frank van Raak, Carla Schölzel-Dorenbos, Desiree Verheijen, Frans Verhey, Marieke Visser, Claire Wolfs, Eddy Adang, Marcel Olde Rikkert

**Affiliations:** 1 Department of Geriatrics/Radboud Alzheimer Centre, Radboud University, Nijmegen Medical Centre, Nijmegen, The Netherlands; 2 Department of Geriatrics, Catharina Hospital, Eindhoven, The Netherlands; 3 Department of Geriatrics, Rijnstate Hospital, Arhem, The Netherlands; 4 Department of Geriatrics, Elkerliek Hospital, Helmond, The Netherlands; 5 Mental Health Organisation Oost-Brabant, centre Land van Cuijk, Boxmeer, The Netherlands; 6 Department of Geriatrics, Slingeland Hospital, Doetinchem, The Netherlands; 7 Department of Geriatrics, Gelderse Vallei Hospital, Ede, The Netherlands; 8 Department of Psychiatry and Neuropsychology/Alzheimer Centre Limburg, Maastricht University Medical Centre+, Maastricht, The Netherlands; 9 Department of Neurology/Alzheimer Centre Amsterdam, VU University Medical Centre, Amsterdam, The Netherlands; 10 Department of Epidemiology, Biostatistics and Health Technology Assessment, Radboud University, Nijmegen Medical Centre, Nijmegen, The Netherlands; Boston Children’s Hospital, United States of America

## Abstract

**Objective:**

To evaluate the cost-effectiveness of post-diagnosis dementia treatment and coordination of care by memory clinics compared to general practitioners’ care.

**Methods:**

A multicentre randomised trial with 175 community dwelling patients newly diagnosed with mild to moderate dementia, and their informal caregivers, with twelve months’ follow-up. Cost-effectiveness was evaluated from a societal point of view and presented as incremental cost per quality adjusted life year. To establish cost-effectiveness, a cost-utility analysis was conducted using utilities based on the EQ-5D. Uncertainty surrounding the incremental cost-effectiveness ratio (difference in costs divided by difference in effects) was calculated by bootstrapping from the original data.

**Results:**

Compared to general practitioners’ care, treatment by the memory clinics was on average €1024 (95% CI: −€7723 to €5674) cheaper, and showed a non-significant decrease of 0.025 (95% CI: −0.114 to 0.064) quality adjusted life years. The incremental cost-effectiveness point estimate from the bootstrap simulation was € 41 442 per QALY lost if one would use memory clinic care instead of general practitioner care.

**Conclusion:**

No evidence was found that memory clinics were more cost-effective compared to general practitioners with regard to post-diagnosis treatment and coordination of care of patients with dementia in the first year after diagnosis.

**Trial Registration:**

ClinicalTrials.gov NCT00554047

## Introduction

The quest for high quality, yet sustainable dementia care is becoming ever more challenging. Dementia is an important – and in numbers growing – cause of disability and burden of care and one of the diseases with the largest per capita healthcare consumption [1], [2]. Moreover, there is a strong trend towards early diagnosis in dementia, which may increase the period during which care for patients with dementia will be asked for [3]. These developments urge to answer the questions of how to optimise care for this population and how to ensure this care for future generations. Trying to answer these questions, several countries have developed national dementia strategies [4], [5], [6], [7], [8]. Many of these strategies focus on the nationwide availability of memory clinics. Therefore, the number of memory clinics in different countries increased rapidly over the last decades [9], [10], [11]. Memory clinics used to focus on diagnosing patients with dementia. Today, memory clinics are also increasingly involved in post-diagnosis treatment and care co-ordination of patients with dementia [9]. There are data supporting the cost-effectiveness of memory clinics as a diagnostic setting [12]. However, evidence about memory clinics being cost-effective in post-diagnosis treatment of dementia and follow-up care is scarce [13]. Knapp and colleagues reviewed the literature on economic evaluations of dementia care [14]. They found that the majority of the economic evidence was on pharmacological interventions. The non-pharmacological interventions, on which they found little economic evidence was often of poor quality and harder to interpret.

Recently we showed that there is no evidence of a difference in effectiveness, evaluated as quality of life of the patient and caregiver burden, between memory clinics and general practitioners with regard to dementia treatment and follow-up care [15]. While – on the basis of these results – effectiveness did not show a significant difference, still performing an economic evaluation of different guidance strategies is relevant, because there may be differences in costs between the two treatment groups justifying implementation of one strategy over the other. To our knowledge, no studies comparing the cost-effectiveness of post-diagnosis dementia treatment and care coordination by memory clinics and general practitioners have been published. Therefore, alongside the randomised trial of which the results of effectiveness were published recently [15], we examined if post-diagnosis treatment and coordination of care for patients with dementia and their caregivers by memory clinics is cost saving and consequently more cost-effective compared to care provided by general practitioners.

## Methods

### Ethical Approval

The study was approved by the Medical Ethics Committee of the Radboud University Nijmegen Medical Centre. Both each patient and informal caregiver gave written informed consent before inclusion in the study.

### Study Design

This study (the AD-Euro Study), was a pragmatic multicentre randomised trial with 12 months’ follow-up. Web based randomisation took place after baseline measurements. Participants (patient-caregiver pairs) were assigned for post-diagnosis dementia care to either the memory clinic or the general practitioner. Details have been published elsewhere [15], [16].

### Participants

The whole study ran from December 2007 until July 2010, with recruitment running from December 2007 until July 2009. Nine Dutch memory clinics recruited participants for whom the diagnostic work up resulted in a new diagnosis of dementia. Each patient had an informal caregiver. Patient-caregiver pairs were excluded when the patient lived in a nursing home, had a life expectancy of less than a year, or needed specific memory clinic care (for example, in the case of Creutzfeldt-Jakob disease) that could not be given by general practitioners.

### Intervention

The interventions consisted of care by either the memory clinic or the general practitioner. The memory clinic provided treatment and care coordination based on the specialist Dutch dementia guideline of the Dutch Institute for Healthcare Improvement [17]. The main content of the intervention of the memory clinic was prescribing and guidance of anti-dementia drugs (cholinesterase inhibitors and memantine). Furthermore, they provided non-drug interventions–for example day structure, referral to a nurse specialist, day care, or home care. According to the guidelines, both drug prescription/guidance and non-drug interventions were delivered on a patient tailored basis. Patient-caregiver pairs assigned to the general practitioner received post-diagnosis treatment and care provided by the general practitioner based on the Dutch general practice and homecare dementia guidelines [18], [19]. Most interventions available in memory clinic care are also available in general practitioner care and were also delivered on a tailored basis.

### Measurements

After baseline measurements, follow-up measurements were made at six and 12 months. Measurements were made at the patients home by interview. Research assistants were instructed, both oral and in writing, how to conduct the interview to prevent differences between interviewers. An overview of the different outcome measures and when they took place has been published elsewhere [16].

#### Outcome measure

To establish cost-effectiveness, a cost-utility analysis was conducted using utilities generated by the EuroQol instrument (EQ-5D) for both patient and caregiver [20], [21], [22]. We used the Dutch utility weight to calculate utilities [23], [24]. From the utility scores Quality Adjusted Life Years (QALYs) were calculated as follows; {[(utility score at baseline+utility score at 6 months)/2] x (6/12)}+{[(utility score at 6 months+utility score at 12 months)/2] x (6/12)}. QALYs of a patient who died during the year of follow-up were given a utility score of zero from their time of death onwards. Costs were calculated from a societal perspective. The incremental cost-effectiveness ratio (ICER) was calculated by dividing the difference in costs by the difference in QALYs. As denominator, we used the sum of the patients and caregiver QALY.

### Costs

Costs were calculated by multiplying volumes of resources by the cost price per resource unit (Table 1). Much of the information about resources used was derived from the Case Report Form (CRF) and was provided by the caregiver. Besides the CRF, we used the hospital information system, the electronic medical record of the general practitioners, and information from different health care workers involved (e.g. physiotherapists, occupational therapists, psychologists) to create the best possible estimate of resources used. We based cost prices on the Dutch guidelines for economic evaluation in healthcare [25], unless stated otherwise. All prices were converted to the year 2009 by means of the consumer price index [26] and expressed in Euros (at the time, €1 was equivalent to British £ 0.85 and US $ 1.43). For a number of resources we linearly interpolated the volume of resources used over the periods between measurements, because no information was available for every single week. The costs for productivity loss of the caregiver were calculated based on the “friction cost method” [25]. The hours of productivity loss were valued at an hourly wage of a cleaning person [25]. With respect to informal care activities, caregivers reported how time spent caring was allocated among activities of daily living (ADLs) and instrumental activities of daily living (IADLs). ADLs involved activities like e.g. dressing, eating, walking, bathing, while IADLs involved more complicated activities like e.g. shopping, providing medication, food preparation or housekeeping [27]. For the time spent on informal care we used the sum of the hours spent on ADLs and IADLs. These hours were valued at an hourly wage of a cleaning person [25].

**Table 1:** Cost variables, resources used, mean price per unit and average costs per patient for the General Practitioner (GP) and Memory Clinic (MC) group.

	Number (%) of patients who used at least one unit of the resource		Average number of used resources during follow-up (total number of units/N)		Mean price (€) per unit (2009)	Mean cost (€) per patient during follow-up (range) (total costs per units/N)	
	MC	GP	MC	GP		MC	GP
	N = 83	N = 77	N = 83	N = 77		N = 83	N = 77
Memory Clinic^¶, §§^					*		
-visits	73 (88%)	4 (5%)	2.4	0.1	69.18	164 (0–415)	5 (0–138)
-telephone	20 (24%)	1 (1%)	0.5	0.03	15.19	8 (0–91)	0 (0–30)
General Practitioner** ^,§§^					*		
-visits	78 (94%)	76 (99%)	5.0	8.1	22.17	115 (0–399)	160 (0–510)
-home visits	48 (58%)	59 (77%)	2.0	2.2	44.33	89 (0–1330)	97 (0–754)
-telephone	51 (61%)	63 (82%)	1.2	1.8	11.08	14 (0–111)	18 (0–78)
Outpatient hospital visits^¶,^ ††	51 (61%)	40 (52%)	1.8	1.7	69.18*	124 (0–830)	118 (0–761)
Medication (average used)^¶,^ **	82 (99%)	74 (96%)	6.3	6.1	variable†	1369 (0–5962)	1287 (0–6784)
Hospital admissions (number of nights)^¶^	16 (19%)	8 (10%)	4.3	0.5	394.22*	1686 (0–48 095)	215 (0–5519)
Home care (hours)^¶^					*		
-domestic care	49 (59%)	49 (64%)	77.2	80.8	16.46	1270 (0–5234)	1330 (0–4329)
-nursing care	34 (41%)	37 (48%)	75.3	83.4	44.33	3339 (0–28 784)	3697 (0–30 645)
Daycare (days)^¶^	31 (37%)	36 (47%)	34.2	37.3	132.78*	4542 (0–52 050)	4946 (0–37 179)
Meals on wheels (days)^¶^	21 (25%)	24 (31%)	46.9	57.1	9.55	448 (0–3630)	546 (0–3983)
Nursing home (days)^¶^	8 (10%)	7 (9%)	7	14.6	226.06*	1585 (0–42 272 )	3300 (0–68 947)
Home for the elderly(days)^¶^	13 (16%)	11 (14%)	36.1	27.5	93.28*	3366 (0–36 844)	2563 (0–37 497)
Physiotherapy (visits)^¶,^ ‡‡	16 (19%)	13 (17%)	5.4	7.9	24.97*	134 (0–2497)	197 (0–2696)
Occupational therapy (visits)^¶,^ ‡‡	4 (5%)	4 (5%)	0.2	0.5	28.40||	5 (0–170)	15 (0–682)
Other professionals^¶,^ ‡‡	14 (17%)	9 (12%)	1.1	0.2	Variable* ^,^ ‡, §, ||	45 (0–1372)	14 (0–254)
Productivity loss (days)^¶^	8 (10%)	3 (4%)	3.4 (N = 32)	0.7 (N = 27)	189.27*	636 (0–11 380)	132 (0–2768)
Informal care (hours)^¶^	77 (93%)	75 (97%)	383	494	9.11*	3487 (0–14 373)	4503 (0–6936)
Total						22 035 (sd 18 800)	23 059 (sd 23 615)

Sources of price information:

* Oostenbrink et al.,

† GIP data/WHO website,

‡ Wolfs et al. §Melis et al.

|| rates of healthcare interventions (http://www.nza.nl/).

Sources of volume information: ¶Case Record Form,

** General practitioner record

†† Electronic Patient File,

‡‡ Health provider, §§ list of interventions.

$ ADL = activities of daily living, IADL = instrumental activities of daily living.

### Statistical Analysis

Data were analysed on an intention-to-treat basis. We used descriptive statistics for baseline characteristics. To present confidence intervals surrounding the costs and effects we undertook nonparametric bootstrapping on the incremental costs and effectiveness with 1000 draws from the original sample. The incremental cost-effectiveness is represented visually by the incremental cost-effectiveness plane. The horizontal axis divides the plane according to incremental effects, whereas the vertical axis divides the plane according to incremental costs. The probability that an intervention is cost-effective varies according to the ceiling ratio. This probability is shown in the cost-effectiveness acceptability curve (CEAC), based on nonparametric bootstrapping [28]. We used Microsoft Office Excel 2007 and SPSS 16.0.01 (release 16.0.2) to do the statistical analyses.

## Results

We included 175 patient-caregiver pairs in the study; 87 were randomly assigned to the memory clinic group and 88 to the general practitioner group. Baseline characteristics of patients and caregivers were similar between the two groups (Table 2). The average age of the patients was 78.1 (SD 5.7) years, and caregivers were on average 63.5 (SD 13.1) years old. The majority of the caregivers (54%, n = 94) were partners, either married or living together with the patient. Most of the patients (60%, n = 105) had Alzheimer’s disease; in 84% (n = 147) of the patients, the severity of the dementia was very mild to mild (clinical dementia rating 0.5 and 1). Cognition of the patients, measured with the mini-mental state examination (range 0–30; higher score indicates better cognition [29]) at baseline was on average 22.7 (SD 3.9). Comorbidity of patients was measured with the cumulative illness rating scale for geriatrics [30]. On average the baseline score was about 9 (range 0–56; higher score indicates more comorbidity). The utility score of the patient at baseline was 0.85 (SD 0.18) in both groups. The utility score of the caregiver was 0.91 (SD 0.15) in the memory clinic group and 0.88 (SD 0.15) in the general practitioner group.

**Table 2:** Baseline characteristics of the participants in the Memory Clinic (MC) group and in the General Practitioner (GP) group.

	MC group (n = 87)		GP group (n = 88)	
	Patient	Caregiver	Patient	Caregiver
Number of participants (n = 175)	87	87	88	88
Female, n (%)	54 (62%)	62 (71%)	52 (59%)	61 (69%)
Age, mean (sd)*	78.2 (6.2)	63.2 (13.4)	77.9 (5.2)	63.9 (12.9)
Type of dementia, n (%)				
Alzheimer’s disease	53 (61%)		52 (59%)	
Vascular dementia	9 (10%)		6 (7%)	
Mixed/other	25 (29%)		30 (34%)	
Severity of dementia, n (%)				
CDR 0,5	3 (3%)		5 (6%)	
CDR 1	70 (81%)		69 (78%)	
CDR 2	14 (16%)		14 (16%)	
Relationship with caregiver, n (%)				
Partner	46 (53%)		48 (55%)	
Child (in law)	36 (41%)		36 (41%)	
Other	5 (6%)		4 (4%)	
MMSE (sd)	22.7 (3.6)		22.7 (4.2)	
CIRS G (sd)	9.2 (4.4)		8.8 (4.6)	
EQ5D-utility (sd)	0.85 (0.18)	0.91 (0.15)	0.85 (0.17)	0.88 (0.15)

* standard deviation, CDR = clinical dementia rating scale (range 0–3; higher score indicates more severe dementia);

CIRS G = cumulative illness rating scale for geriatrics (range 0–56; higher score indicates more comorbidity); MMSE = mini-mental state examination (range 0–30; higher score indicates better cognition).

### Outcome

We evaluated 160 pairs (77 in the general practitioner group and 83 in the memory clinic group) out of the 175 patient-caregiver pairs included in the study. Eleven pairs (four in the memory clinic group) dropped out because they considered further participation to be too burdensome. One caregiver died, one caregiver did not fill out the questionnaires, and one caregiver was not present during the measurements without giving any reasons, all in the general practitioner group. In one patient in the general practitioner group the diagnosis of dementia was changed just after inclusion and was the reason for the patient and caregiver to withdraw from the study.

The average cost, cumulated over 12 months follow-up, was €22 035 (range €682 to €120 698) per patient in the memory clinic group. In the general practitioner group the mean cost per patient was €23 059 (range €674 to €78 721). This resulted in a statistically not significant difference between the two study-groups of €1024 (95% CI: −€7723 to €5674). The analyses showed a difference in QALYs of 0.025 in favour of the general practitioner group, which was also not statistically significant (95% CI: −0.114 to 0.064). Including the minimisation factors as covariates in the regression analysis left the difference in costs between the two study-groups (€2441 (95% CI: −€8152 to €3270)) and the difference in QALYs (0.029 (95% CI: −0.1 to 0.06)) unchanged. Also, adding comorbidity as covariate, left the difference between the two study-groups unchanged (difference in costs €3470 (95% CI −€9257 to €2316) and in QALYs 0.015 (95% CI: −0.1 to 0.07)). From the different cost variables (Table 1) three variables were significant different between the memory clinic and the general practitioner group; the costs of hospital admissions (p = 0.03), contact with the general practitioner (p = 0.03) and contact with the memory clinic (p = 0.00).

**Figure 1:**
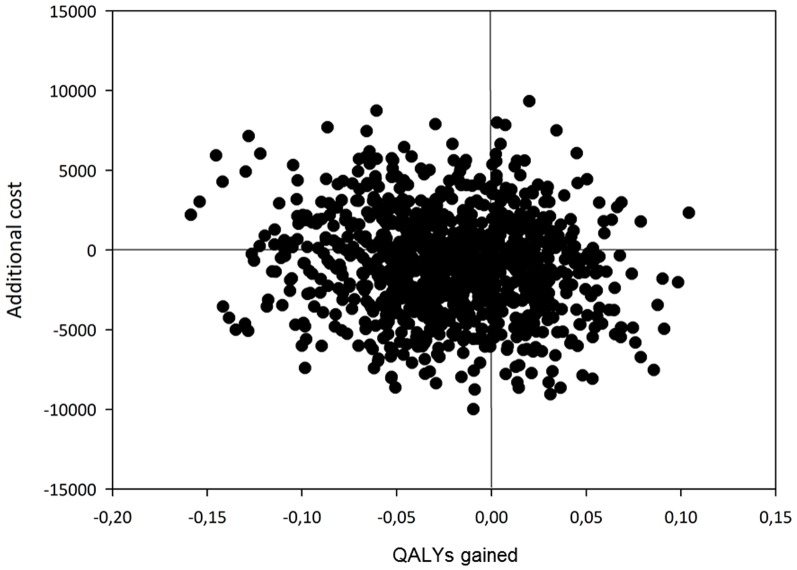
Scatterplot of the estimated incremental costs and incremental effects obtained by bootstrap simulations.

The incremental cost-effectiveness point estimate from the bootstrap simulation was € 41 442 per QALY lost if one would use memory clinic care instead of general practitioner care. The 95% confidence interval surrounding the incremental cost-effectiveness ratio (cost per QALY gained) in the bootstrap simulation ranged from −€1 221 001 (2.5 percentile) to €1 026 234 (97.5 percentile), using the percentile method. The scatter plot in Figure 1 shows the visual representation of the result of the bootstrap simulation. 59% of the bootstrapped ICERs is situated below the horizontal axis (x-axis), meaning that the majority of the ICERs indicate that the treatment in the memory clinic is cheaper than for the general practitioner. Further, 66% of the simulations is situated left from the vertical axis (y-axis), meaning that a majority of the simulated ICERs indicate that the general practitioner is more effective than the memory clinic. As a second scenario we used the UK utility weights to calculate the incremental cost-effectiveness ratio in a bootstrap simulation. The results were similar to the results shown here and therefore not shown.

**Figure 2:**
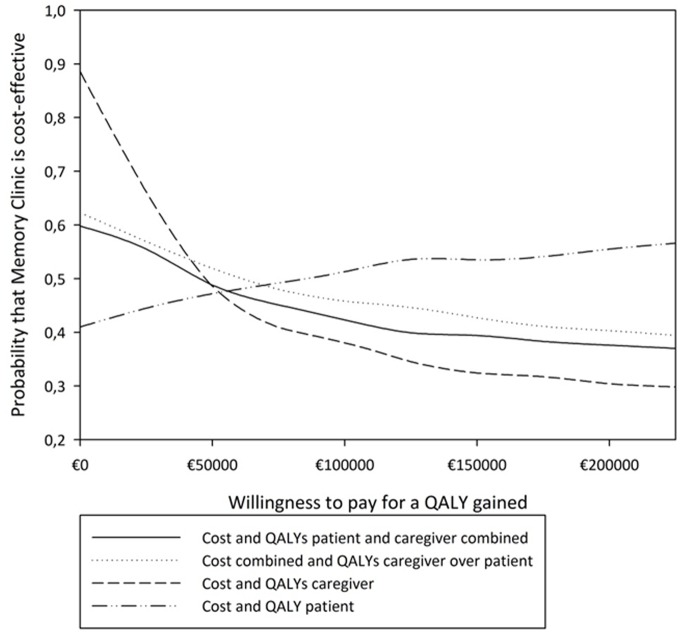
Cost-effectiveness acceptability curve. The probability that the Memory Clinic was cost-effective compared with the General Practitioner (solid line). The dotted line shows the curve if only the QALY of the patient as rated by the caregiver was used. The striped and the stripe-dot line show the probability if the cost and QALY of the patient alone and if the cost and QALY of the caregiver alone (cost of the caregiver being caregiving time and productivity loss) were used respectively.

The cost-effectiveness acceptability curve for the situation of our primary cost-effectiveness analysis (ICERs numerator: all costs for both patient and caregiver, denominator: the QALY’s of the patients and their caregivers combined) is shown in Figure 2 by the solid line. This figure indicates what would be an acceptable amount of money to compensate the loss of a QALY if one would choose memory clinic care over general practitioner care. For example if a monetary compensation of €50 000 for one lost QALY would be acceptable, then the probability of cost-effectiveness is similar for both modalities (general practitioner and memory clinic). If a smaller monetary value for a lost QALY is acceptable, then the probability that the memory clinic is more cost-effective increases to approximately 60%. In this last scenario there will be no monetary compensation at all for a lost QALY. Figure 2 also shows that the results are very much identical if we used the QALY of the patient as rated by the caregiver instead of the sum of the QALY of a patient-caregiver pair (dotted line). Using only patients QALY and costs the probability that the memory clinic is more cost-effective increases if a greater amount of money than a monetary compensation of €50 000 for a QALY gained would be acceptable. It can be noticed from Figure 2 that all CEACs decrease as a function of the money value for a QALY except the CEAC based on the patient alone.

## Discussion

We found, applying a societal point of view, no evidence of a statistical significant difference in cost-effectiveness between memory clinics and general practitioners with regard to post-diagnosis treatment and coordination of care for patients with dementia at one year follow-up. A Bayesian interpretation of cost-effectiveness (the CEAC) also shows that cost-effectiveness between both modalities remains unresolved.

### Comparison with other Studies

Comparison of our results with others is difficult, because of the lack of similar studies. The study of Wolfs et al. showed that, in comparison with usual care, an integrated multidisciplinary diagnostic approach to dementia in a memory clinic setting increased the health related quality of life of dementia patients and was cost-effective [12]. Compared with our study, their patients’ cognition was worse and the patients had a much lower mean quality adjusted life year value, which could be reasons why we found relatively low costs in our study compared to the study by Wolfs and colleagues.

Other studies compared cost of care of specific interventions – which could be part of post diagnosis dementia guidance – with alternatives [14]. Evidence for cost-effectiveness was seen for certain pharmacological treatments and for some selected non-pharmacological interventions. For example Graff and colleagues found that occupational therapy for patients with dementia and their caregivers was cost-effective compared with usual care [31]. However, cost-effectiveness data for the comparison between general practitioner and memory clinic concerning post-diagnostic care were not available.

### Strengths and Limitations

We ensured a robust study design by using a randomised controlled trial and we carried out the economic evaluation from a societal perspective. By using a generic measure for quality of life (EQ-5D) we are able to compare our results with other interventions. This is important to interpret the cost savings for a QALY lost. The participation of nine different memory clinics, based in different settings (university hospital, general hospital, old age psychiatry), enhanced the heterogeneity among care setting and thus the generalisability of our study results. However, differences in healthcare systems and variability in dementia care between countries makes international generalisability of our results difficult. Furthermore it is argued that, especially when effects are expressed as QALYs in an economic evaluation, there is a risk of double counting. We based QALYs on the EQ-5D where productivity is no dimension. Therefore double counting seems less of a threat than if quality of life was measured on for example a VAS (Visual Analogue Scale), ranging from best to worst imaginable quality of life. Further Krol et al. suggest that QALY measures such as the EQ-5D are insensitive to concerns regarding effects on income even when these are (explicitly) incorporated [32].

Another limitation that possibly may have affected the outcome of this study is the length of the follow-up period. Dementia is a disease that progresses over years, so an extended follow-up lasting several years would be preferable to the relatively short 12 month period we used. It is known that costs increase over time in case of a patient with dementia.

The lack of difference in costs was unanticipated due to the assumption that primary care in general is cheaper. A possible explanation could be that our group of patients was in their first year after diagnosis with relatively mild dementia and therefore often could take care of oneself with relatively little help from others, both in the memory clinic and in the general practitioner group. However, this is a very topical subject as there is a strong international trend towards early diagnosis of dementia. Nevertheless, this means that these results cannot be generalised to more severely affected individuals. With further progress of the disease other needs will occur and it could be that in the long term a difference between the memory clinic and the general practitioner group could emerge.

### Implications and Conclusions

This study adds important data, which seem to point to a lack of difference in cost-effectiveness between memory clinics and general practitioners in the treatment and coordination of care for patients with dementia at one year follow-up. Together with former results that no evidence was found of memory clinics being more effective than general practitioners [15], these data are very important for the ongoing debate on which type of post-diagnosis treatment and follow-up care is best for which patients. The development of dementia plans in many countries and the just recently published report of the World Health Organisation has once more stressed the need to continue to compare different strategies of dementia care and to continue to increase the evidence base [7], [33], [34]. If further studies verify our results, this indicates that memory clinic guidance on average is not more efficient than general practitioners in these early stages of dementia. This is an important message, for both patients-caregivers and policy makers, especially as one has to anticipate to the trend of early diagnosis, and make the best choices for the future. Under the scenario of lacking superiority of memory clinics, both in terms of effectiveness and costs, other factors should determine the most sustainable and efficient post-diagnosis dementia care.

As our follow-up period was only one year after diagnosis, using a decision analysis modelling strategy to compare costs and effectiveness of the interventions in longer term should be a topic for future research.
